# Supplementary figures and images for: Interference Underlies Attenuation upon Relearning in Sensorimotor Adaptation
Source: eNeuro. 2025 Jun 18;12(6):ENEURO.0132-25.2025. doi: 10.1523/ENEURO.0132-25.2025 (PMC12203768; doi:10.1523/ENEURO.0132-25.2025)

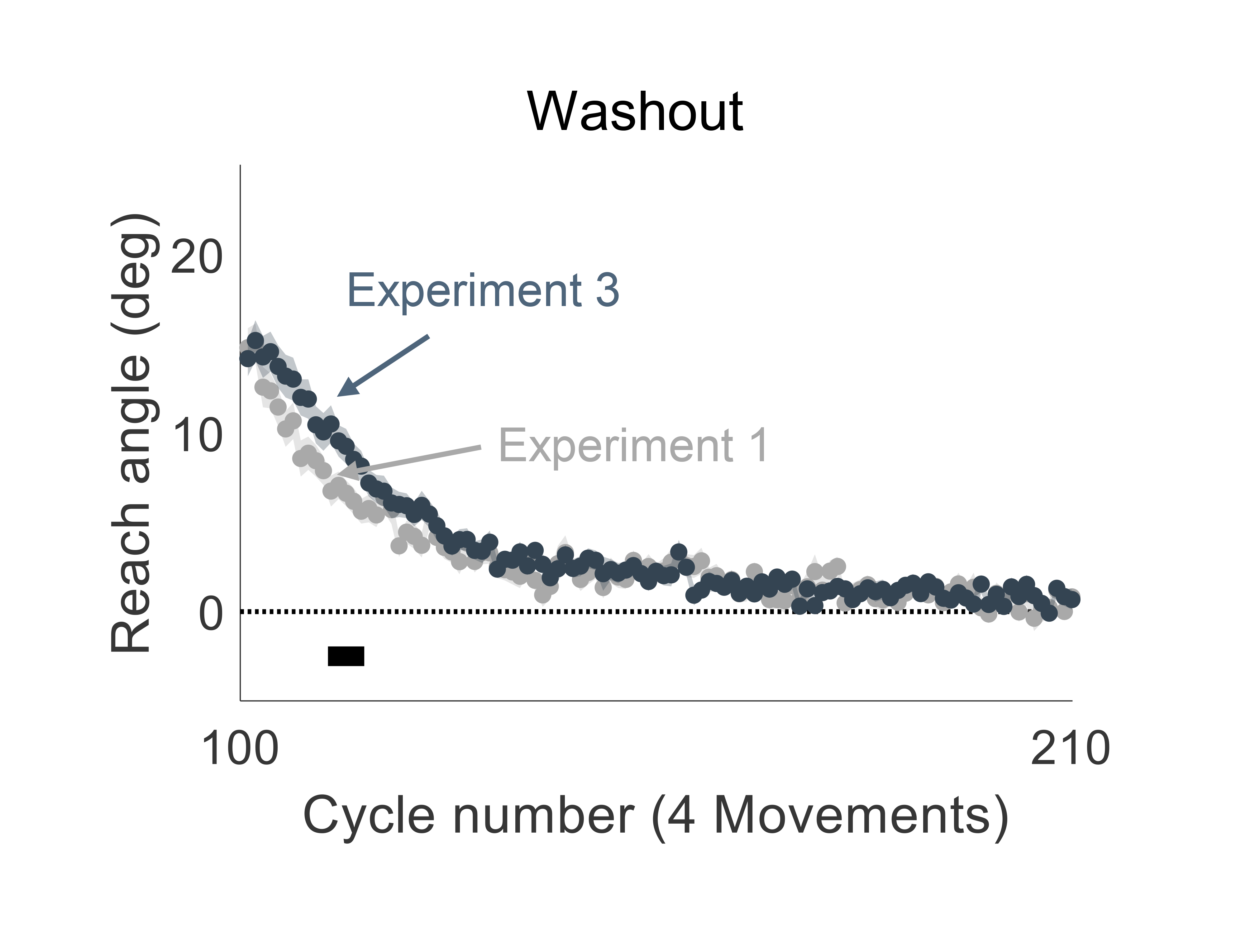

Supplement: Figure 3-1 — Early washout in response to a contingent visuomotor rotation that decreases in a gradual manner (Experiment 3) was slower than washout driven by a reversed clamp (Experiment 1). Overlaid time courses of mean reach angle during the washout block for Experiment 1 (gray) and Experiment 3 (blue), with the data averaged within each cycle of four movements. The horizontal black bar during the early phase of the washout block denotes a cluster that showed a significant difference between the two experiments, suggesting that the rate of de-adaptation during washout was lower in response to the gradual, contingent rotation schedule that we used compared to a reversed clamp. Download Figure 3-1, TIF file. [file eneuro-12-ENEURO.0132-25.2025-s002.tif]

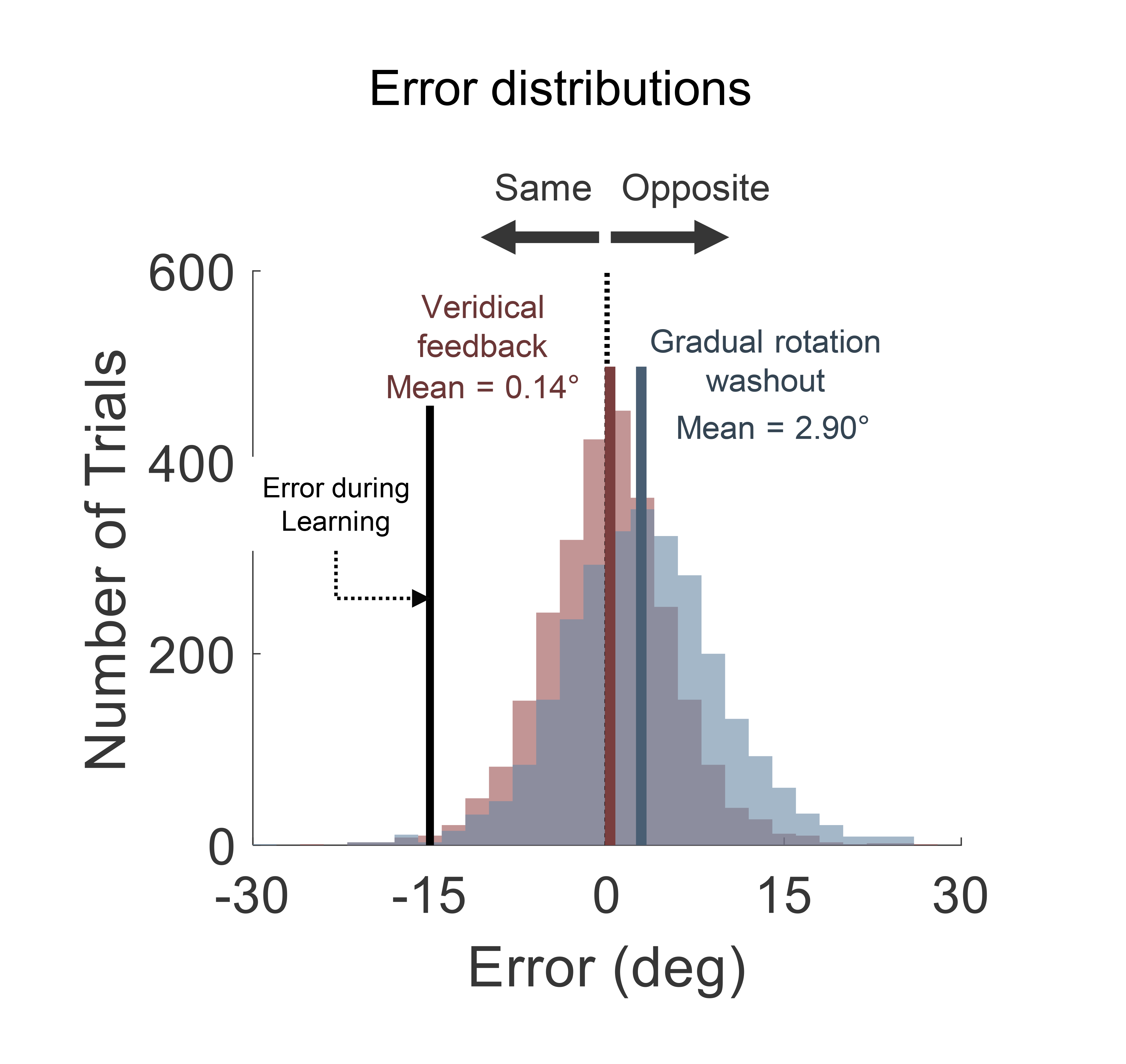

Supplement: Figure 3-2 — Comparison of error distributions observed in response to the gradual rotation washout or veridical feedback. Overlaid distributions of errors experienced during the non-zero rotation phase of the washout block in Experiment 3 ([mean ± standard deviation], 2.90° ± 10.1°, blue) and with veridical feedback (0.14° ± 10.6°, red). The latter data was obtained from a different group of participants (N = 44) which experienced a 195-cycle block with veridical feedback. We used the data from cycles 100-116, matching the phase of the experiment and the mean number of trials used for calculating the distribution in Experiment 3. The distribution from Experiment 3 is biased such that most errors experienced in this phase of the washout block are in the opposite direction of the error experienced during the initial learning block (black solid line). Presumably, these opposite errors are the signals that drive the washout of the initial adaptation. The dotted line represents zero error. Download Figure 3-2, TIF file. [file eneuro-12-ENEURO.0132-25.2025-s003.tif]
